# 3-oxo-C12:2-HSL, quorum sensing molecule from human intestinal microbiota, inhibits pro-inflammatory pathways in immune cells via bitter taste receptors

**DOI:** 10.1038/s41598-022-13451-3

**Published:** 2022-06-08

**Authors:** Garance Coquant, Doriane Aguanno, Loïc Brot, Christine Belloir, Julie Delugeard, Nathalie Roger, Hang-Phuong Pham, Loïc Briand, Marielle Moreau, Luisa de Sordi, Véronique Carrière, Jean-Pierre Grill, Sophie Thenet, Philippe Seksik

**Affiliations:** 1 Sorbonne Université, INSERM, Centre de Recherche Saint-Antoine, 75012 Paris, France; 2 Paris Center for Microbiome Medicine (PaCeMM) FHU, APHP, Paris, Ile-de-France France; 3 EPHE, PSL University, 75014 Paris, France; 4 Centre des Sciences du Goût et de l’Alimentation, UMR 1324 INRAE, UMR 6265 CNRS, University of Bourgogne Franche-Comté, 21000 Dijon, France; 5 Parean Biotechnologies, Saint Malo, France; 6 LVMH Recherche, Life Science Department, 45800 Saint Jean de Braye, France; 7 Département de Gastroentérologie et Nutrition, APHP, Hôpital Saint-Antoine, Sorbonne Université, 75012 Paris, France

**Keywords:** Cell biology, Cell signalling, Inflammatory bowel disease, Inflammation, Microbial communities

## Abstract

In the gut ecosystem, microorganisms regulate group behaviour and interplay with the host via a molecular system called quorum sensing (QS). The QS molecule 3-oxo-C12:2-HSL, first identified in human gut microbiota, exerts anti-inflammatory effects and could play a role in inflammatory bowel diseases where dysbiosis has been described. Our aim was to identify which signalling pathways are involved in this effect. We observed that 3-oxo-C12:2-HSL decreases expression of pro-inflammatory cytokines such as Interleukine-1β (− 35%) and Tumor Necrosis Factor-α (TNFα) (− 40%) by stimulated immune RAW264.7 cells and decreased TNF secretion by stimulated PBMC in a dose-dependent manner, between 25 to 100 µM. Transcriptomic analysis of RAW264.7 cells exposed to 3-oxo-C12:2-HSL, in a pro-inflammatory context, highlighted JAK-STAT, NF-κB and TFN signalling pathways and we confirmed that 3-oxo-C12:2-HSL inhibited JAK1 and STAT1 phosphorylation. We also showed through a screening assay that 3-oxo-C12:2-HSL interacted with several human bitter taste receptors. Its anti-inflammatory effect involved TAS2R38 as shown by pharmacologic inhibition and led to an increase in intracellular calcium levels. We thus unravelled the involvement of several cellular pathways in the anti-inflammatory effects exerted by the QS molecule 3-oxo-C12:2-HSL.

## Introduction

Inflammatory bowel disease (IBD), including Crohn’s disease (CD) and ulcerative colitis (UC), are chronic relapsing inflammatory conditions of the gastrointestinal tract leading to bowel damages and increased risk of intestinal cancer^1^. The incidence and prevalence of CD and UC are increasing and an estimated 0.3% of the European population suffers from IBD, representing 2.5–3 million people. IBD, which affects young adults and is long-lasting, accounts for substantial costs to the health care system and society. Disease management has changed significantly over the last decade with the use of parenterally administrated biologic agents and new small oral molecules such as pan-Janus kinase (JAK) inhibitor^2^. Immunosuppressants are used more widely and earlier in the progression of the disease. However, this strategy is not always effective, is quite expensive and exposes patients to adverse effects, mainly serious infections and malignancies^3^. Thus, there is both a need and a place for more “gut restricted” and physiological therapeutic approaches mostly based on a better knowledge of the gut microbiota.

As a matter of fact, gut microbiota has been strongly implicated in the pathogenesis of IBD^4,5^ where microbial unbalance (dysbiosis) has been described^6^. Thus microbiota-based treatments, such as faecal microbiota transplantation, are currently being studied^7,8^. In this setting, investigating gut microbiota-derived molecules remains an attractive strategy to control gut inflammation and prevent IBD flares. In fact, gut microbiota reciprocally interacts with co-evolved host epithelial and immune cells. This results in a beneficial mutualistic relationship in which microbiota synthesizes metabolites that can modulate the host’s immune responses^9–12^.

Diffusible signal compounds represented by bacterial quorum sensing (QS) molecules, called autoinducers have attracted increasing interest due to their effects on host cells demonstrated in various models^13–15^. Acyl-Homoserine Lactones (AHL) are a class of autoinducers that can impact the human host, as part of the inter-kingdom signalling^16^. Our team identified for the first time several AHLs in the gut ecosystem, and among them, the most prominent was the 3-oxo-C12:2-HSL, a molecule that had never been described^17^. 3-oxo-C12:2-HSL was decreased in IBD patients compared to healthy subjects and its presence was correlated with normobiosis^17^. 3-oxo-C12:2-HSL is structurally close to 3-oxo-C12-HSL synthesized by *Pseudomonas aeruginosa,* a molecule whose effects on host cells are well reported in the literature^15,16^. We previously described the anti-inflammatory and dose-dependent effects of 3-oxo-C12:2-HSL on intestinal epithelial cells Caco-2/TC7 stimulated by interleukin-β^17^. In addition, we have demonstrated that 3-oxo-C12:2-HSL protects against tight junctions disruption induced by pro-inflammatory cytokines and thus has a beneficial role on intestinal barrier function^18^. In the present study, we aim to characterise the effects of 3-oxo-C12:2-HSL on immune cells and to identify cellular targets of the autoinducer. Using the macrophage cell line RAW264.7 as well as peripheral blood mononuclear cells (PBMC) stimulated by lipopolysaccharides (LPS) and interferon-γ (IFNγ), we assessed how 3-oxo-C12:2-HSL modulates host-inflammatory response and the mechanisms involved. Our results show that this AHL exerts anti-inflammatory effects by down-regulating pro-inflammatory cytokines secretion. Using transcriptomic analysis, we have identified several pathways involved, one of them being the JAK-STAT pathway. 3-oxo-C12:2-HSL is able to prevent RAW264.7 macrophages from JAK-STAT signalling activation induced by LPS and IFNγ. Additionally, based on the interaction of 3-oxo-C12-HSL from *P. aeruginosa* with bitter taste receptor TAS2R38^19^, we demonstrated that several bitter taste receptors are potential membrane receptors for 3-oxo-C12:2-HSL.

## Results

### 3-oxo-C12:2-HSL reduces expression and secretion of pro-inflammatory cytokines by activated immune cells

**Figure 1:**
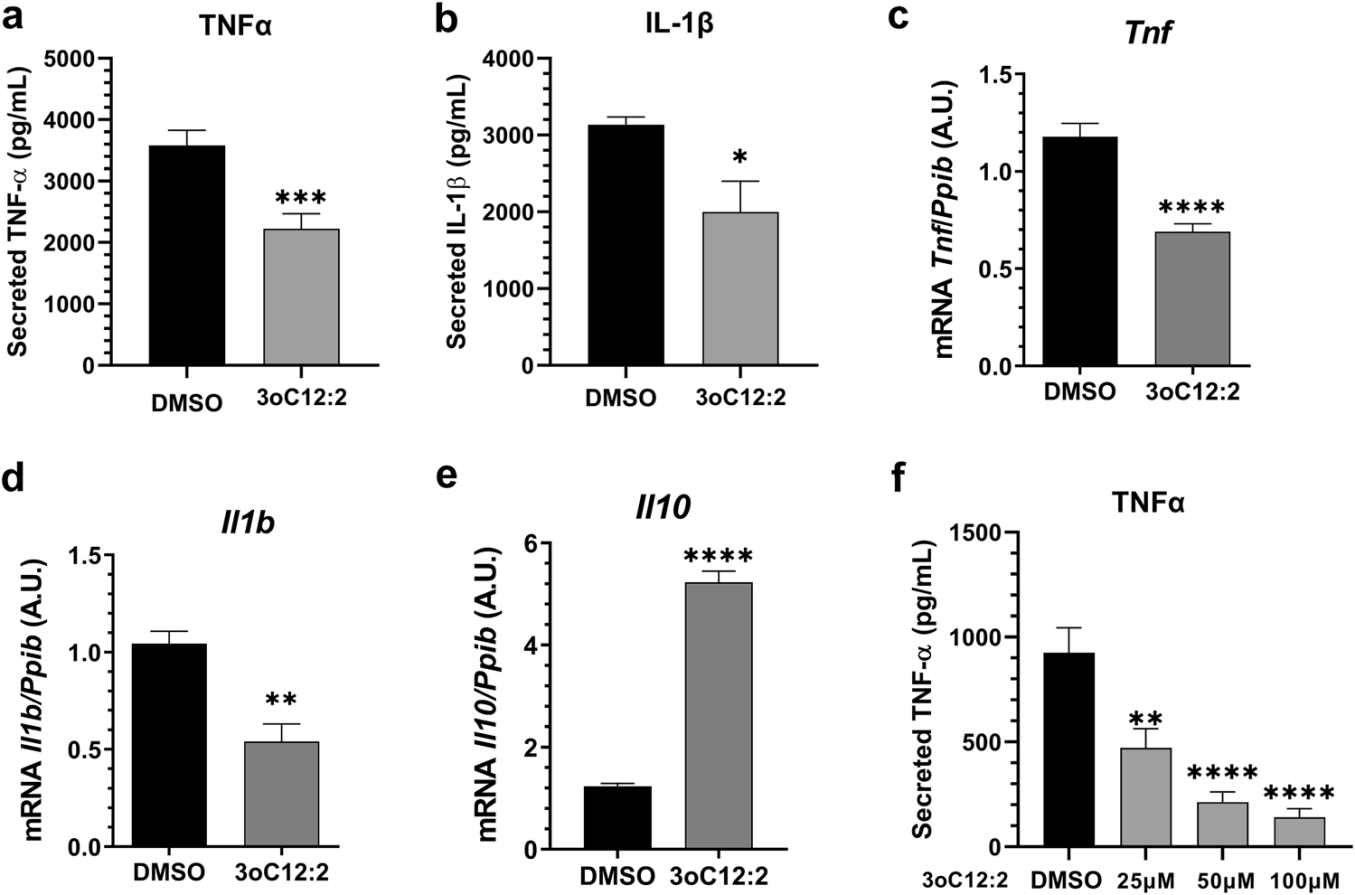
3-oxo-C12:2-HSL decreases pro-inflammatory cytokine secretion by immune cells. Secreted TNFα (**a**) and IL-1β (**b**) levels were measured by ELISA from supernatant of RAW264.7 macrophages stimulated with LPS and IFNγ in absence (control) or presence of 50 µM 3-oxo-C12:2-HSL for 6 h. Unpaired *t* test, *p ≤ 0.05, **p < 0.01, ***p < 0.001, ****p < 0.0001 vs*.* control. mRNA levels for *Tnf* (**c**), *Il1b* (**d**), *Il10* (**e**)*,* genes measured by RT-qPCR in RAW264.7 macrophages stimulated with LPS and IFNγ in absence (control) or presence of 50 µM 3-oxo-C12:2-HSL for 2 h. Results are expressed in arbitrary units as a ratio of the target gene to cyclophilin (*Ppib*) used as housekeeping gene. Unpaired *t* test, *p ≤ 0.05, **p < 0.01, ***p < 0.001, ****p < 0.0001 vs. control. (**f**) Secreted TNFα measured by ELISA from supernatant of PBMC stimulated with LPS (10 ng/mL) and exposed to a concentration range of 3-oxo-C12:2-HSL (0–100 µM). Results are expressed as mean ± SEM of triplicates from 3 independent experiments. One Way ANOVA Dunnett’s post-test. 3oC12:2 stands for 3-oxo-C12:2-HSL.

We first observed the effect of 3-oxo-C12:2-HSL on cytokine secretion by immune cells. We exposed RAW264.7 murine macrophages cells to lipopolysaccharides (LPS) and Interferon-γ (IFNγ) to trigger classical activation of M1 macrophages^20^. Cells were treated with or without 50 µM of 3-oxo-C12:2-HSL followed by analysis of cytokines levels in the supernatant (Fig. 1a,b). Lower secretion of pro-inflammatory Tumor Necrosis Factor-α (TNFα) (− 40%, Fig. 1a) and Interleukine-1β (IL-1β) (− 35%, Fig. 1b) was observed in presence of 3-oxo-C12:2-HSL compared with the control. We then investigated the effects on inflammation at the transcript level. mRNA levels of the two cytokines mentioned above were reduced in activated macrophages exposed to 3-oxo-C12:2-HSL, to the same extent as the protein (Fig. 1c,d). Anti-inflammatory cytokine IL-10 expression was strongly increased when cells were exposed to the AHL (Fig. 1e).

To confirm these results in a more physiological model, we tested the impact of the AHL on Peripheral Blood Mononuclear Cells (PBMC) from healthy donors. Inflammation was triggered with LPS (10 ng/mL), and we exposed PBMC to an increasing dose of 3-oxo-C12:2-HSL. We showed that TNFα secretion was strongly decreased in a dose-dependent manner, between 25 to 100 µM (Fig. 1f).

Of note, cytotoxicity was monitored through lactate dehydrogenase release for all experiments and there was no significant difference compared to the control for the doses of AHL used (Supplementary Fig. 1).

### 3-oxo-C12:2-HSL modulates gene expression in RAW264.7 cells

**Figure 2:**
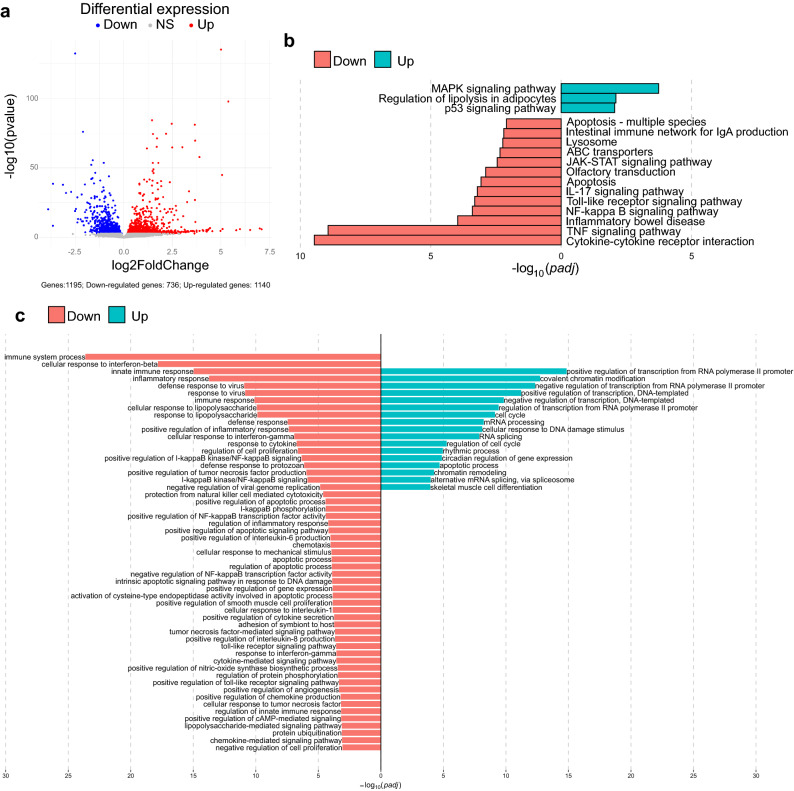
3-oxo-C12:2-HSL modulates gene expression in RAW264.7 macrophage cells. Cells were stimulated with LPS and IFNγ in absence (control) or in presence of 50 µM 3-oxo-C12:2-HSL for 2 h. (**a**) Volcano plot of differentially expressed genes between the activated cells cultured in absence and in presence of 3-oxo-C12:2-HSL. Blue, grey and red dots represent down regulated, not significantly regulated and up regulated genes respectively. (**b**) Significant KEGG pathways involved in the inflammation process and differentially modulated by 3-oxo-C12:2-HSL were identified by EGSA method (p < 0.01). Red and green bars represent down-regulated and up-regulated pathways respectively. All significant modulated pathways are displayed in Supplementary Fig. 2. (**c**) Significant Gene Ontology (GO) biology processes enriched by differentiated expressed genes modulated by 3-oxo-C12:2-HSL. Terms were annotated using the Database for Annotation, Visualization and Integrated Discovery (DAVID). Red and green bars represent down-regulated and up-regulated pathways respectively. *GO* Gene Ontology, *KEGG* Kyoto Encyclopedia of Genes and Genomes.

In an attempt to understand the mechanisms underpinning the immunomodulatory effects of 3-oxo-C12:2-HSL on immune cells, we performed a transcriptomic analysis of the activated RAW264.7 cells exposed to 3-oxo-C12:2-HSL. We observed that 3-oxo-C12:2-HSL down-regulated 736 genes and up-regulated 1140 genes compared with control cells treated with DMSO (Fig. 2a).

Differentially expressed genes were annotated by Gene Ontology (GO) analysis (Fig. 2c). GO analysis revealed that down-regulated genes were enriched in “immune system process”, “innate immune response” and “inflammatory response” categories, in accordance with the anti-inflammatory effects of 3-oxo-C12:2-HSL observed in Fig. 1. In terms of molecular function, we identified that the downregulated genes were enriched in the NF-κB and Toll Like Receptor (TLR) and TNF signalling pathways. The latter correlated with the decrease of TNFα protein secretion observed in Fig. 1a,f.

A KEGG pathway analysis was carried out to classify and group differentially expressed genes in cellular processes. A large number of pathways were modulated by the AHL (Supplementary Fig. 2), among which, the most down-regulated pathways were “Cytokine–cytokine receptor interaction” and “TNF signalling pathway”. A selection of pathways related to inflammation and IBD modulated by 3-oxo-C12:2-HSL is displayed on Fig. 2b. This includes crucial pathways directly involved in cell signalling during inflammation such as the “JAK-STAT signalling pathway”, the “NF-κB signalling pathway” and the “TNF signalling pathway”. This also includes pathways related to IBD such as “apoptosis” and “ABC transporters”. Interestingly, 3-oxo-C12-HSL produced by *P. aeruginosa* was reported to up-regulate both apoptosis and expression of ABC receptors^21^, suggesting that these two AHL do not exert similar effects.

From both GO and KEGG pathway analyses, we were able to pinpoint specific cellular pathways involved in the signalling of 3-oxo-C12:2-HSL. As JAK-STAT pathway is shared by several cytokine receptors and known to mediate key deleterious effects of pro-inflammatory cytokines in IBD^22,23^, we further investigated it in presence of 3-oxo-C12:2-HSL.

### 3-oxo-C12:2 prevents the activation of the JAK-STAT pathway

**Figure 3:**
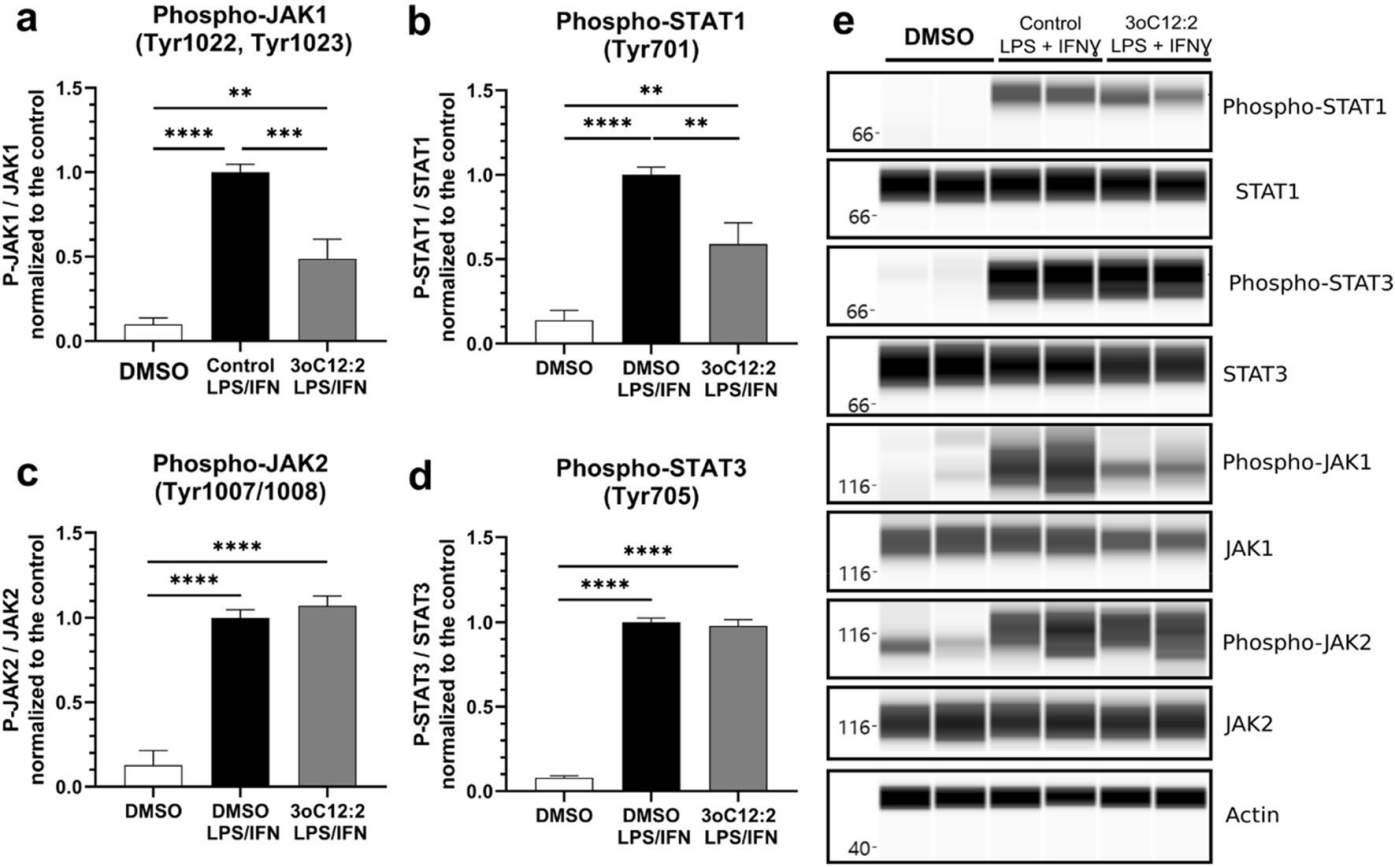
3-oxo-C12:2-HSL prevents activation of the JAK-STAT pathway. Cells were stimulated with LPS and IFNγ in absence (control) or in presence of 50 µM 3-oxo-C12:2-HSL for 2 h. The levels of phosphorylated proteins P-JAK1 (**a**), STAT1 (**b**), JAK2 (**c**), STAT3 (**d**) were normalized to their respective unphosphorylated forms. Results are expressed as mean ± SEM from 4 independent experiments. One-Way ANOVA, Dunnett’s post-test **p < 0.01, ***p < 0.001, ****p < 0.0001 vs. control. (**e**) Reconstructed images from Simple Western analysis of protein levels and actin used as housekeeping protein are displayed. They are based on the area under the chemiluminescence signal curve obtained for one experiment, representative of 4 independent experiments performed in duplicates. Molecular markers are indicated on the left (kDa). 3oC12:2 stands for 3-oxo-C12:2-HSL.

To explore the effects of 3-oxo-C12:2-HSL on the JAK-STAT pathway, we analysed the phosphorylation level of proteins involved in this pathway. As the latter is composed of a family of JAK and STAT proteins, we studied several of them in RAW264.7 macrophages activated by LPS and IFNγ and exposed or not to 3-oxo-C12:2-HSL (Fig. 3). When cells were treated with the pro-inflammatory cocktail, we observed an increase of the phosphorylation of JAK1, JAK2, STAT1, STAT3 proteins (Fig. 3a–e). When activated macrophages were exposed to 3-oxo-C12:2-HSL, a reduced phosphorylation level of JAK1 (− 50%) and STAT1 (− 40%) protein was observed, in comparison with activated cells without AHL treatment (Fig. 3a,b). However, JAK2 and STAT3 phosphorylation levels were not significantly altered upon exposure to 3-oxo-C12:2-HSL.

These results demonstrate that 3-oxo-C12:2-HSL prevents the activation of the JAK-STAT pathway, by specifically reducing the phosphorylation of JAK1 and STAT1 proteins.

### 3-oxo-C12:2-HSL interacts with bitter taste receptors

It was previously reported that the AHL 3-oxo-C12-HSL synthesized by *P. aeruginosa* might exert some of its effects through its interaction via the human bitter taste receptor TAS2R38^19,24^. Bitter taste receptors are G protein-coupled receptor and their role beyond oral taste has been extensively studied throughout the last decade^25^. Taste receptors are all expressed along the digestive tract^26–29^, but also by immune cells^19,30,31^. Based on the similarities in the chemical structure of 3-oxo-C12-HSL and 3-oxo-C12:2-HSL, we adopted a targeted approach to identify potential eukaryotic receptors for intestinal 3-oxo-C12:2-HSL and hypothesized that 3-oxo-C12:2-HSL could also interact with the bitter taste receptor TAS2R38.

**Figure 4:**
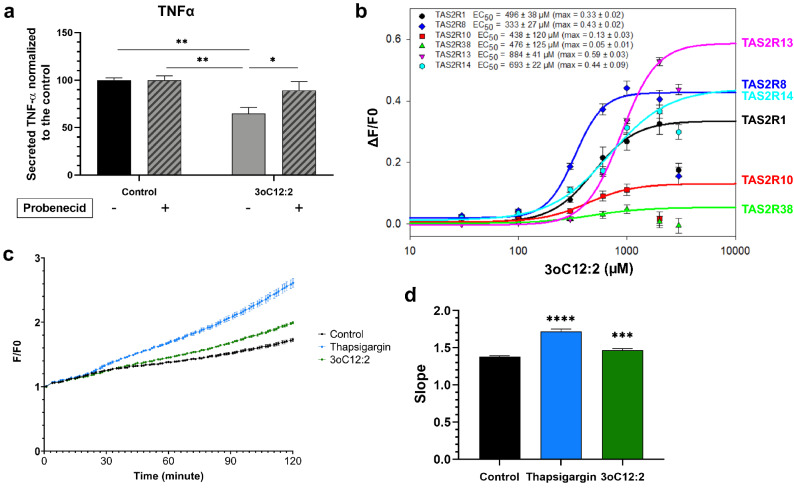
3-oxo-C12:2-HSL interacts with bitter taste receptors. (**a**) RAW264.7 macrophages were pre-treated or not with 1 mM Probenecid, an allosteric inhibitor of the bitter taste receptor TAS2R138 one hour before their stimulation with LPS and IFNγ in absence (control) or in presence of 3-oxo-C12:2-HSL for six hours. Mean ± SEM from 3 independent experiments performed in triplicate, Two Way ANOVA Tukey’s post-test. (**b**) Calcium response in HEK293T cells expressing reporter human bitter taste receptor exposed to several concentrations of 3-oxo-C12:2-HSL (0–1000 µM). Fluorescence (F) was measured after exposure to the AHL and normalized to basal fluorescence (F0). Mean ± SEM from 3 independent experiments performed in triplicate. (**c**) Intracellular calcium flux in RAW264.7 cells exposed to 1000 µM 3-oxo-C12:2-HSL or 10 µM thapsigargin as a positive control or DMSO (0.5%) as a negative control. Fluorescence (F) monitoring the increase in intracellular concentration of calcium was measured for two hours and normalized to basal fluorescence (F0). Each curve is representative of 3 independent experiments of 8 replicates. (**d**) Quantification of the slope of the curves displayed in (**c**). Values are mean ± SEM of 3 independent experiments (8 replicates each). For (**a**) and (**d**): Two Way ANOVA Tukey’s post-test. *p ≤ 0.05, **p < 0.01, ***p < 0.001, ****p < 0.0001 vs. control. 3oC12:2 stands for 3-oxo-C12:2-HSL.

We pre-exposed RAW264.7 cells to probenecid, a known allosteric inhibitor of TAS2R38^32,33^ and its mouse orthologue TAS2R138 then added LPS and IFNγ and 3-oxo-C12:2-HSL (Fig. 4a). First, we noted that probenecid treatment had no effect on the TNFα secretion in activated cells cultured in absence of 3-oxo-C12:2-HSL (control). When exposed to probenecid and 3-oxo-C12:2-HSL in a pro-inflammatory context, more TNFα was secreted compared to cells only exposed to 3-oxo-C12:2-HSL without inhibitor treatment. This result shows that when TAS2R138 is inhibited, the 3-oxo-C12:2-HSL anti-inflammatory effect is abolished.

To elucidate if 3-oxo-C12:2-HSL was able to activate human TAS2R38 and other bitter taste receptors, we screened 25 known human bitter taste receptors in HEK293T reporter-cells (Fig. 4b)^34^. Among these bitter taste receptors, the AHL was able to activate TAS2R1, TAS2R8, TAS2R10, TAS2R13, TAS2R14, and TAS2R38. TAS2R13 displayed the highest maximum amplitude (max ampl = 0.59 ± 0.03), while having the highest half maximal effective concentration (EC_50_ = 884 ± 41 µM). TAS2R1, TAS2R8 and TAS2R14 showed similar maximum amplitude values (around 0.45), but TAS2R8 was the most sensitive to 3-oxo-C12:2-HSL (EC_50_ = 333 ± 27 µM), making it a good candidate for further analysis. TAS2R10 and TAS2R38 exhibited similar EC_50_ values (EC_50_ = 438 ± 120 µM and 476 ± 125 µM respectively) but had much lower amplitude responses (max ampl = 0.13 ± 0.03 and 0.05 ± 0.01 respectively).

Transduction of taste signalling results in the opening of IP3R-dependant calcium channels and the intracellular Ca^2+^ release^25^. Therefore, we investigated the effect of 3-oxo-C12:2-HSL with a Fluo-4 probe in the basal state, using the endoplasmic reticulum Ca^2+^ ATPase inhibitor thapsigargin as a positive control. We observed an increase in intracellular calcium level when RAW264.7 cells were exposed to 3-oxo-C12:2-HSL compared with the control (Fig. 4c,d). The quantification of calcium response shows that in presence of 3-oxo-C12:2-HSL the slope of the curve was significantly higher than that observed in the control condition (Fig. 4d).

## Discussion

As the human gut is one of the most densely populated microbial ecosystems, studying QS molecules, related to bacterial density, seems legitimate in this environment. AHLs are the most studied QS auto-inducers and, using mass spectrometry, our team previously identified one that had never been documented before in the gut ecosystem, 3-oxo-C12:2-HSL^17^. Our initial work showed that 3-oxo-C12:2-HSL correlated with normobiosis and was less prevalent in IBD associated dysbiosis.

**Figure 5:**
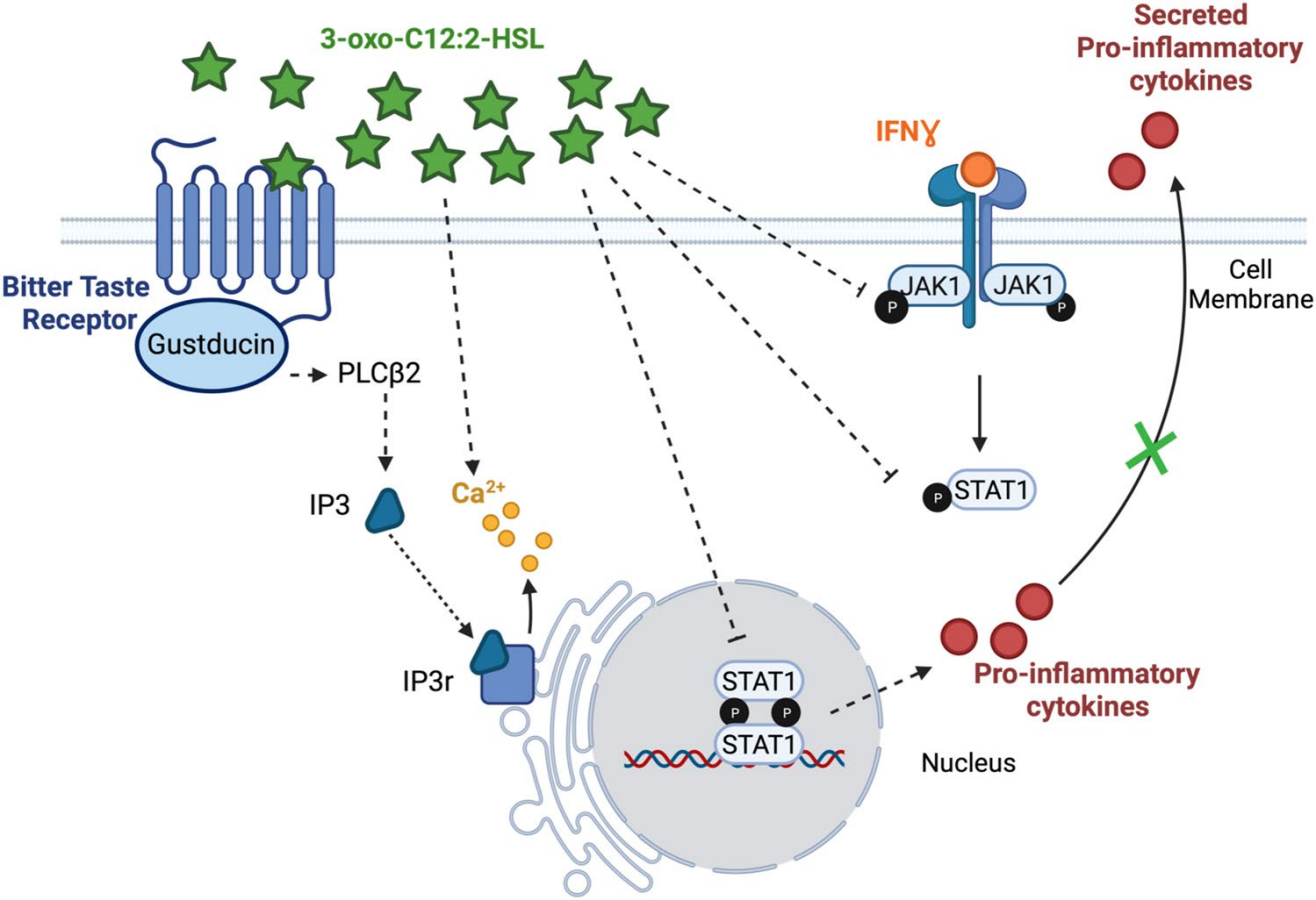
Proposed mechanisms of 3-oxo-C12:2-HSL effects on inflammation. In immune cells, 3-oxo-C12:2-HSL is able to activate bitter taste receptors, which are G-protein coupled receptors, triggering a signalling cascade resulting in the release of calcium from the endoplasmic reticulum. In addition, in murine activated macrophages the presence of 3-oxo-C12:2-HSL attenuates the activation of the JAK-STAT signalling pathways, by specifically preventing JAK1 and STAT1 phosphorylation. This leads to a decrease in pro-inflammatory cytokine secretions and an overall reduced inflammatory response. Part of the effects of 3-oxo-C12:2-HSL on inflammatory response is dependent on bitter taste receptors. Created with BioRender.com.

Our group observed that 3-oxo-C12:2-HSL decreased IL-8 production in the intestinal epithelial cell line Caco-2/TC7 stimulated by IL-1β^17^. We therefore sought to investigate how 3-oxo-C12:2-HSL can modulate inflammatory response of immune cells. Here, we demonstrate that 3-oxo-C12:2-HSL exerts anti-inflammatory effects on immune cells by preventing JAK-STAT pathway activation and by activating bitter taste signalling (Fig. 5).

AHLs have been described to modulate host’s immunity, in particular 3-oxo-C12-HSL from *Pseudomonas aeruginosa*, structurally close to 3-oxo-C12:2-HSL. A number of studies have reported that 3-oxo-C12-HSL downregulated cytokine secretion by macrophages^35–37^, dendritic cells^38^, T lymphocytes^35,39,40^ as well as epithelial cells^17,41^. In the present study, we show that the intestinal unsaturated 3-oxo-C12:2-HSL displays anti-inflammatory effects by downregulating secretion of pro-inflammatory cytokine TNFα and IL-1β, as well as their respective gene expression by RAW264.7 murine macrophages. Interestingly, this AHL was also able to increase anti-inflammatory cytokine IL-10 expression, suggesting a potent anti-inflammatory effect in immune cells. These findings were confirmed using a more physiological model, PBMC stimulated by LPS, in which TNFα was strongly decreased by 3-oxo-C12:2-HSL exposure, in a dose-dependent manner. We activated RAW264.7 cells with LPS and IFNγ, leading the macrophages to a M1 profile^20^, similar to what is observed during IBD physiopathology. Indeed, in the mucosa of patients, there is an influx of pro-inflammatory macrophages^42^. Under such conditions, activation of the JAK-STAT pathway is enhanced, with a particular involvement of the STAT1 protein^43–45^. One study previously reported that 3-oxo-C12-HSL is able to modulate the JAK-STAT pathway in breast carcinoma cells^46^, but our group is the first to report AHL modulation of this pathway in immune cells. 3-oxo-C12:2-HSL was able to prevent the phosphorylation of JAK1 and STAT1, which is particularly relevant in the field of IBD. Indeed, 3-oxo-C12:2-HSL, which is decreased in IBD patients^17^, appears as a microbial product able to modulate IBD pathways involved in chronic inflammation. Novel IBD treatments are targeting the JAK proteins^23^. Current IBD drugs target one or more cytokines (such as anti-TNF)^23^ while new oral small molecules act by inhibiting either pan-JAK or specific JAK pathways. However, clinical trials with JAK inhibitors revealed serious adverse events^23^. Alternative approaches using molecules targeting the gut are appealing, with drugs exerting a strong anti-inflammatory effect while being less prone to side effects. The use of AHL as modulators of gut inflammation is thus an interesting and promising path. Moreover, 3-oxo-C12:2-HSL was shown to stabilize tight junctions proteins involved in gut barrier^18^, thereby counterbalancing the disruption of the gut barrier in IBD^47^. Altogether, these results pave the way for future therapeutic development of this type of molecule. In this setting, it remains crucial to study the mechanisms of action by which a given AHL signals its effect on epithelial and immune cells. Our future studies will focus on the JAK-STAT pathway and explore how 3-oxo-C12:2-HSL is able to prevent phosphorylation of JAK1 and STAT1 proteins.

Interkingdom effects of AHL raise the question of eukaryotic AHL receptors. Some proteins were identified as target of 3-oxo-C12-HSL from *P. aeruginosa*: the scaffolding protein IQGAP1f., the aryl hydrocarbon receptor (AhR)^48^, PPARγ^49,50^, the bitter taste receptor TAS2R38^19,24,51,52^. The latter has aroused our curiosity due to the growing interest in bitter taste receptors, particularly in their extra-oral expression^25^. In this study, we demonstrate that TAS2R38 signalling is needed for 3-oxo-C12:2-HSL to exert anti-inflammatory effects on immune cells. Taste receptor signalling, through the second messenger IP3, activates calcium release from the endoplasmic reticulum^25^. We demonstrated that 3-oxo-C12:2-HSL is able to induce calcium flux in RAW264.7 macrophages, thus reinforcing the hypothesis that 3-oxo-C12:2-HSL signals through taste receptors. Furthermore, through a screening of all human bitter taste receptors, we identified six taste receptors that were activated in the presence of 3-oxo-C12:2-HSL. The six receptors displayed different response amplitudes and maximal effective concentration, with TAS2R8 being the most sensitive (lowest EC50) to the AHL. Some of the tested BTR have numerous known ligands, such as TAS2R14 which can bind to more than 150 compounds, while others only have two known synthetic agonists like TAS2R13, according to the BitterDB database^53^. Among the receptors we identified as activated by 3-oxo-C12:2-HSL, TAS2R8, TAS2R10 TAS2R13 and TAS2R14 are closely related, while TAS2R1 and TAS2R38 are more distant (Supplementary Fig. 3). BTR seem to share high homology in their intra-membrane and intra-cytoplasm domains but their extra-cellular domain is more diverse, possibly to allow the recognition of several agonists^34^. A study described precisely the interactions between AHLs and BTR at the amino-acid level, and showed that the AHLs tested bind to the same extra-cellular orthosteric site on different BTR (TAS2R4, TAS2R14, TAS2R20)^24^. Future structure–function studies on those identified domains could allow determining their involvement in the interaction with 3-oxo-C12:2-HSL. Advances in knowledge of BTR involvement in inflammatory pathways are needed to link our findings with AHLs signalling in immune cells.

In the present study, we restricted our investigation to the effect of 3-oxo-C12:2-HSL on host immune cells. One could imagine that 3-oxo-C12:2-HSL also exerts an effect on gut microbiota. Since we have previously shown that the absence of 3-oxo-C12:2-HSL is associated with the differential representation of several bacterial taxa in faecal microbiota from IBD patients^17^, we could hypothesise that 3-oxo-C12:2-HSL participates in gut normobiosis.

Gut dysbiosis is now a well-recognized feature in IBD. This condition can promote or inhibit specific bacteria species of interest, which have been demonstrated to play a role in the pathophysiology^54^. Therefore, elucidating the origin of 3-oxo-C12:2-HSL in the intestine would allow a major advance in understanding its possible involvement in microbiota eubiosis and the physiology of the host. Several teams have attempted to use a bioinformatics approach to determine a system of bacterial synthesis of AHLs in the intestine^17,55–58^. Among the genes of the microbiome that have been sequenced and listed in the databases, no solid lead has been highlighted and such investigations have to be pursued. Concerning specifically 3-oxo-C12:2-HSL, it could be the product of a trophic chain, where host enzymes would be involved, in particular to generate the double unsaturations.

It is worth noting that in the complex gut ecosystem, several QS auto-inducers coexist and may have opposite effects. Most effects exerted by QS molecules are detrimental to the host; for instance 3-oxo-C12-HSL from *P. aeruginosa* disrupts barrier function^15,18^, AI-3 synthesized by enterohemorrhagic *E. coli* exerts pro-inflammatory effects on host-cells^59^ and the universal QS molecule AI-2 produced by *Fusobacterium nucleatum* promotes pro-inflammatory macrophages^60^. On the opposite, a study showed that AI-2, delivered by modified *E. coli*, increased Firmicutes proportions of the microbiota following an in vivo antibiotic challenge and was able to recapitulate gut microbiota^61^. In the same way, the role of 3-oxo-C12:2-HSL in shaping gut microbiota needs to be examined. Its putative effects induced in the gut microbiota, which must be investigated, could be additive to its effects on host cells, leading to an overall beneficial effect in gut homeostasis.

Our work is part of an innovative approach highlighting the role of bacterial QS molecules in the gut apart from the interaction with a pathogen. Our results provide a first set of data on the interactions of the gastrointestinal tract with AHL and we propose a model laying the foundation for the effect of 3-oxo-C12:2-HSL on immune cells (Fig. 5). 3-oxo-C12:2-HSL is able to activate bitter taste receptors, resulting in calcium release from the endoplasmic reticulum. Besides, 3-oxo-C12:2-HSL prevents from the activation of the JAK-STAT pathways in activated macrophages by specifically preventing the phosphorylation of JAK1 and STAT1. Finally, the effect of 3-oxo-C12:2-HSL on immune cells highlights the role of this type of molecule in the pathophysiology of puzzling diseases such as IBD where dysbiosis is involved in gut inflammation. Investigating QS molecules and their interkingdom effect therefore remains an unavoidable pursuit when searching for links between inflammation and gut microbiota.

## Methods

### Cell culture

RAW264.7 murine macrophage cells (ATCC, Manassas, Virginia, USA) were cultured between passage 12 and 25 in Dulbecco’s modified Eagle’s medium supplemented with 10% heat-inactivated fetal calf serum and 1% l-glutamine (ThermoFisher Scientific). The macrophages were seeded at 35,000 cells per well in 12-well plates (Falcon) for ELISA experiments or 110,000 cells per well in 6-well plates (Falcon) for cell lysates, upon reaching 80–90% confluence after 3-day culture. Cells were maintained at 37 °C with 5% CO2/air atmosphere and the media were changed every other day.

### Human peripheral blood mononuclear cell isolation

Peripheral Blood Mononuclear Cells (PBMC) were extracted from whole blood of anonymous donors and collected by *Etablissement Français du Sang* of Paris, France.

PBMC were cultured in Roswell Park Memorial Institute medium (ThermoFisher Scientific) with 10% heat-inactivated fetal calf serum, 1% l-glutamine, 1% non-essential amino acids, 1% sodium pyruvate, penicillin (100 IU/mL), and streptomycin (100 µg/mL). Peripheral blood was harvested in EDTA tubes, blood was diluted with PBS 1:1, layered over Histopaque-1077^®^ (Sigma) and centrifuged for 20 min at 400*g* at room temperature. Extracted PBMC were washed twice with sterile PBS then centrifuged at 300*g* for 5 min and resuspended in complete medium. For experiments, cells were seeded at 300,000 cells per well in 200 µL of medium in 96-well plates (ThermoFisher Scientific).

### Cell treatments

Cells were exposed to 3-oxo-C12:2-HSL (synthesized as previously described^17^) or DMSO 0.1% as a control for different durations depending on the experiment, in the presence of the lactonase inhibitor 2-hydroxyquinoline (100 µM, Sigma-Aldrich), in order to limit the hydrolysis of the AHL lactone ring, as described previously^17,18^.

In RAW264.7 cells, inflammation was triggered with LPS (10 ng/mL, Sigma) and IFNγ (10 U/mL, R&Dsystems) for 2 h or 6 h, depending on the experiments. PBMC were exposed to LPS (10 ng/mL) and AHL for 24 h. For TAS2R38 inhibition experiments, cells were exposed to TAS2R38 allosteric inhibitor probenecid (1 mM, Sigma) for one hour before adding AHLs and the inflammatory cocktail, without changing the media.

### Lactate dehydrogenase release

Release of lactate dehydrogenase into the medium was measured to assess cellular toxicity. LDH concentration was measured using the Cytotoxicity Detection Kit (Roche, Boulogne-Billancourt, France) according to the manufacturer’s instructions. The media were centrifuged 5 min at 250*g* to remove any cells. 50 µL of reaction mixture was added to 50 µL of media supernatant in 96-well plates. After 10 min at room temperature, 25 µL of stop solution was added. Absorbance at 490 nm was determined with a microplate spectrometer (FLUOstar Omega; BMG Labtech). Results are expressed as absorbance arbitrary units.

### Enzyme-linked immunosorbent assay

At the end of cell treatment, the medium was centrifuged at 300*g* for 5 min at 4 °C. Supernatant was frozen at − 80 °C until ELISA assays. TNFα and IL-1β ELISA kits were purchased from R&D systems and performed according to the manufacturer’s instructions. Briefly, plates were coated with a capture antibody and incubated overnight. Wells were blocked using 1% bovine serum albumin-PBS solution (Sigma), then exposed to cell supernatants at room temperature for two hours. Wells were washed and incubated with the detection antibody for an additional two hours. After thorough washing, Streptavidin-HRP solution was added for 20 min. Wells were washed, then incubated with TMB (Biolegend) solution for 20 min and the reaction was stopped using 1 M H_2_SO_4_. Absorbance was measured by a spectrometer (FLUOstar Omega; BMG Labtech) at 450 nm and the absorbance at 540 nm was subtracted.

### RNA extraction for RNA sequencing

Cells were treated for 2 h and then washed once with PBS before RNA extraction using RNeasy minikit (Qiagen), according to the manufacturer’s instructions. Briefly, cells were lysed and 1 volume of ethanol was added. The sample was transferred into a spin column and centrifuged at 8000*g* for 15 s. The column was washed several times and then eluted with water. RNA quality was assessed using NanoChip (Agilent) on a chip reader (Bioanalyzer 2100, Agilent).

### mRNA sequencing

After extraction, total RNA were qualified with the AGILENT tapeStation 2200. Preparation of mRNA libraries was performed according to the manufacturer’s recommendations (KAPA mRNA HyperPrep Kit from ROCHE). Final samples pooled into a library were sequenced on ILLUMINA Novaseq 6000, corresponding to 2 × 28 Millions of 100 bases reads per sample after demultiplexing.

### RNA-Seq data analysis workflow

FastQC v 0.11.9^62^ was used for the quality control of raw paired-end fastq data set. Illumina’s adapters were removed from reads using cutadapt v2.10^63^. Trim_galore v0.6.4^64^ was used to trim bad quality bases (Phred < 20). Salmon v 1.4.0^65^ was used to quantify paired-end reads against a mapping-based index built from the Ensembl GRCm38 transcript set for all samples. Downstream analysis was handled in R platform v4.0.2^66^ and its add-on packages. Quantified transcripts were imported into R using the package tximport v1.16.1^67^. Gene-level DESeqDataSet object was built from previously imported transcript level abundances using the package DESeq2^68^ to perform the differential expression analysis. A pre-filtering was applied to remove genes with less than 5 counts in one sample. For visualisation, raw counts were transformed using the “variance stabilizing transformation” method implemented in the package DESeq2. For an overview of the variability among samples, Principal Component Analysis was performed on variance stabilizing transformation and normalised count dataset using the package ade4 v 1.7-16^69^. Differential expression analysis was carried out by the DESeq function of the DESeq2 package. Briefly, the size factor was estimated for each sample, the dispersion was estimated for each gene, counts were fitted to a Negative Binomial Generalized Linear Model and Wald’s test was conducted to test the difference in expression between biological conditions. The Benjamini–Hochberg method^70^ was used for controlling the False-discovery rate (FDR). Significance level was fixed at type I error alpha = 0.01. Volcano plots were used to display the results of differential analysis using the ggplot2 v3.3.3^71^ package. All significant gene lists were annotated for enriched biological functions and pathways using the DAVID platform through the RDAVIDWebService v1.28.0^72^ package for gene ontology^73^ and Kyoto Encyclopedia of Genes and Genomes terms^74^. Significant terms had adjusted p-value, according to the Benjamini–Hochberg method, below 0.05. In addition to the traditional gene by gene analysis, count dataset was analysed at KEGG pathway level using the package Ensemble of Gene Set Enrichment Analysis (EGSEA) v1.16.0^75^ that combines results from twelve algorithms to improve the biological relevance of pathways. Thus, significant pathways had adjusted p-value below 0.01. Gene expression heatmaps were produced by the pheatmap v1.0.12^76^ package. We used Venn diagrams to globally visualize the overlap between all significant genes as well as all significant pathways in the biological condition comparisons.

### Determination of protein levels by Wes™ capillary electrophoresis

RAW264.7 cells were scraped in lysis buffer (20 mM Tris- HCl, pH 7.4, 5 mM EDTA, 0.15 M NaCl, 1% Triton X-100, 0.5% sodium deoxycholate) supplemented with protease inhibitor cocktail (Complete Mini; Roche, Boulogne-Billancourt, France) and phosphatase inhibitor cocktail (PhosSTOP, Sigma-Aldrich). Protein concentrations were determined using the BC Assay (Uptima/Interchim). Wes™ analyses (capillary electrophoresis system; ProteinSimple, San Jose, CA) were performed according to the manufacturer’s recommendations, and adequate protein concentrations and antibody dilutions were determined in preliminary assays to allow optimal quantitative conditions. The microplates were loaded with 0.8 μg/μL protein, primary antibodies (as detailed below), and reagents provided by the manufacturer: anti-JAK1 (1:25; Biotechne MAB4260), anti-Phospho-JAK1 (1:100; Invitrogen 44422G), anti-JAK2 (1:13; Cell Signalling, 3230), anti-Phospho-JAK2 (1:13; Cell Signalling 3776), anti-STAT1 (1:50; Cell Signalling 9172), anti-Phospho-STAT1 (1:40; Biotechne AF2894), anti-Actin (1:1000; Millipore MAB1501R). Data were analysed using Compass for SW3.1 software (ProteinSimple). Protein levels were determined by chemiluminescence signal (AUC) and Phospsho-Protein levels were normalized to the total level of each protein of interest, after verification that actin levels do not differ between conditions.

### Bitter taste receptors screening

Bitter taste receptor cDNA were cloned into the pcDNA4 mammalian expression vector (Invitrogen) using full coding receptor sequences reported on UniProt database. The synthetic constructs were codon optimized (Genewiz) and combine the first 45 amino acids of rat somatostatin type 3 receptor at the amino terminus to improve plasma membrane targeting of the receptors in the heterologous system^77^. The FLAG epitope was added to the carboxy terminus without interfering with receptor functionality and can be used for immunocytochemistry. Calcium-mobilization assays were performed using human embryonic kidney cells (HEK293T) stably transfected with chimeric Gα16gust44 protein^78^. Cells were seeded in black, poly-d-lysine coated 96-well plates at a density of 35,000 cells/100 µL in high glucose DMEM supplemented with 10% dialysed fetal bovine serum and 1% penicillin/streptomycin. Twenty-four hours later, the cells were transiently co-transfected using Lipofectamine 2000 (Life Technologies) with one of the TAS2R synthetic optimized vectors or empty expression plasmid, as a negative control, and the pCMV-GCaMP5G construct (Addgene #31788) coding for a genetically encoded calcium indicator^79,80^. Twenty-four hours after transfection, cells were washed twice with C1 buffer (130 mM NaCl, 5 mM KCl, 10 mM HEPES, 2 mM CaCl2, 5 mM sodium pyruvate, pH7.4). 3-oxo-C12:2-HSL was solved in DMSO and further diluted in C1 buffer. Next, the cells were placed in a fluorometric imaging plate reader (Flexstation^®^ 3, Molecular Devices) and stimulated with automatic injection of increasing concentrations of AHLs. Calcium responses leading to increase in fluorescence were monitored at 510 nm after excitation at 488 nm. Experiments were performed in duplicates and repeated at least four times. The recorded calcium levels of each wells receiving the same stimulus were averaged, response of mock-transfected cells were subtracted from receptor-transfected cells and net signals were normalized to background fluorescence (F/F0, F0 fluorescence light before stimulus application). The resulting dose–response curves of the averaged fluorescent signal amplitudes against the logarithm of the agonist concentration were fitted using Sigma Plot software. For data analysis, the four-parameter logistic equation [f(x) = min + (max − min)/(1 + (x/EC_50_)nH)] was used to calculate the half-maximal effective concentrations (EC_50_ values).

### RNA extraction and RT-qPCR

Total RNA was extracted from RAW264.7 cells using TRIzol (Invitrogen) according to the manufacturer's instructions. Reverse Transcription (RT) was performed with 1 μg RNA using LunaScript^®^ RT SuperMix Kit (NewEngland Biolabs). Semi-quantitative real-time PCR was performed with the Mx3000P Stratagene system using SYBR Green (Applied Biosystems) according to the manufacturer's procedures. Cyclophilin was used as the reference gene. After amplification, Ct were determined. Sequences of the oligonucleotide primers used are reported in Table 1.

**Table 1:** Sequences of the primers used in this study.

Target gene (protein)	Forward primer	Reverse primer
*Tnf* (TNFα)	CCAGACCCTCACACTGAGATC	CACTTGGTGGTTTGCTACGAC
*Il1b* (IL-1β)	AGTTGACGGACCCCAAAAG	AGCTGGATGCTCTCATCAGG
*Il10* (IL-10)	CTGGACAACATACTGCTAACC	GGGCATCACTTCTACCAGGTA
*Ppib* (Cyclophilin B)	GCCTTAGCTACAGGAGAGAA	TTTCCTCCTGTGCCATCTC

### Intracellular calcium assay

RAW264.7 cells were seeded in black, clear-bottom 96-well plates (Costar) at 20,000 cells per well. The next day, release of intracellular Ca^2+^ was measured using Fluo-4 (Fluo-4 Direct Calcium Assay; Life Technologies) according to the manufacturer’s instructions. Medium was discarded and 100 µL of 1 X Fluo-4 dye was incubated at 37 °C for 1 h in 5% CO_2_, protected from light. Then, basal fluorescence was measured using a fluorospectrometer (FLUOstar Omega; BMG Labtech), excitation at 485 nm and emission at 520 nm. Molecules of interest were then added with an electronic pipette; thapsigargin (10 µM, Sigma) as a positive control, 3-oxo-C12:2-HSL (1000 µM) or DMSO 0.5%; with 8 replicates per conditions for every experiment. The fluorescence signal was immediately measured every 80 s for 2 h. Results are expressed as a ratio of the fluorescence x times over the basal fluorescence (F/F0).

### Quantification and statistical analysis

Data are expressed as means ± standard error of the mean (SEM) unless otherwise indicated. The number of independent experiments and replicates are indicated in the figure captions. Figures and statistical analyses were performed with Graphpad^®^ Prism 8.0 (Ritme Informatique, Paris, France). Normal distribution was tested using Shapiro–Wilk test. When normal distribution was confirmed, statistical differences between the means were assessed by ANOVA or *t* test otherwise by a non-parametric Kruskal–Wallis test. Post-test are indicated in the figure captions. For all results, *p* < 0.05 was considered statistically significant. Values are represented as follows: *p ≤ 0.05; **p < 0.01; ***p < 0.001; ****p < 0.0001.

### Ethics declarations

Peripheral Blood Mononuclear Cells (PBMC) were extracted from whole blood of anonymous donors and collected by *Etablissement Français du Sang* of Paris, France. Informed consent was obtained from all participants.

## Supplementary Information

Supplementary Figures.

## Data Availability

The datasets generated and/or analysed during the current study are available from the corresponding author upon reasonable request.
