# Supplementary material for: 3-oxo-C12:2-HSL, quorum sensing molecule from human intestinal microbiota, inhibits pro-inflammatory pathways in immune cells via bitter taste receptors
Source: Sci Rep. 2022 Jun 8;12:9440. doi: 10.1038/s41598-022-13451-3 (PMC9177545; doi:10.1038/s41598-022-13451-3)
Supplement: Supplementary file 1 — Supplementary Figures. [file 41598_2022_13451_MOESM1_ESM.pdf]

3-oxo-C12:2-HSL, Quorum Sensing molecule from human intestinal microbiota, inhibits pro-inflammatory pathways in immune cells *via* bitter taste receptors

Garance Coquant[1,2], Doriane Aguanno[1,2,3], Loïc Brot[1,2], Christine Belloir[4], Julie Delugeard[1,2], Nathalie Roger[1,2], Hang-Phuong Pham[5], Loïc Briand[4], Marielle Moreau[6], Luisa De Sordi[1,2], Véronique Carrière[1,2], Jean-Pierre Grill[1,2], Sophie Thenet[1,2,3], Philippe Seksik[1,2,7]*

[1]Sorbonne Université, INSERM, Centre de Recherche Saint-Antoine, CRSA, F-75012 Paris, France [2] Paris Center for Microbiome Medicine (PaCeMM) FHU, APHP, Paris, Ile-de-France, France [3]EPHE, PSL University, Paris, France, [4]Centre des Sciences du Goût et de l'Alimentation, UMR 1324 INRAE, UMR 6265 CNRS, University of Bourgogne Franche-Comté, F-21000 Dijon, France, [5]Parean Biotechnologies, Saint Malo, France, [6]LVMH Recherche. Life Science Department, 185 Avenue de Verdun, 45800. Saint Jean de Braye. France [7]Sorbonne Université, Département de Gastroentérologie et Nutrition, APHP Hôpital Saint-Antoine, F-75012 Paris, France

Supplementary figure 1

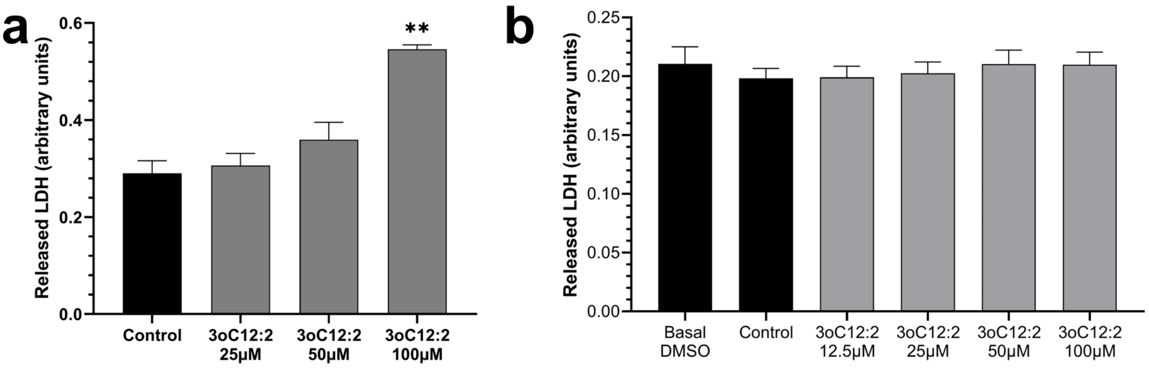

Supplementary Figure 1: LDH release by (a) RAW264.7 murine macrophages and (b) human peripheral blood mononuclear cell exposed to a range of concentration of 3-oxo-C12:2-HSL

Supplementary figure 2

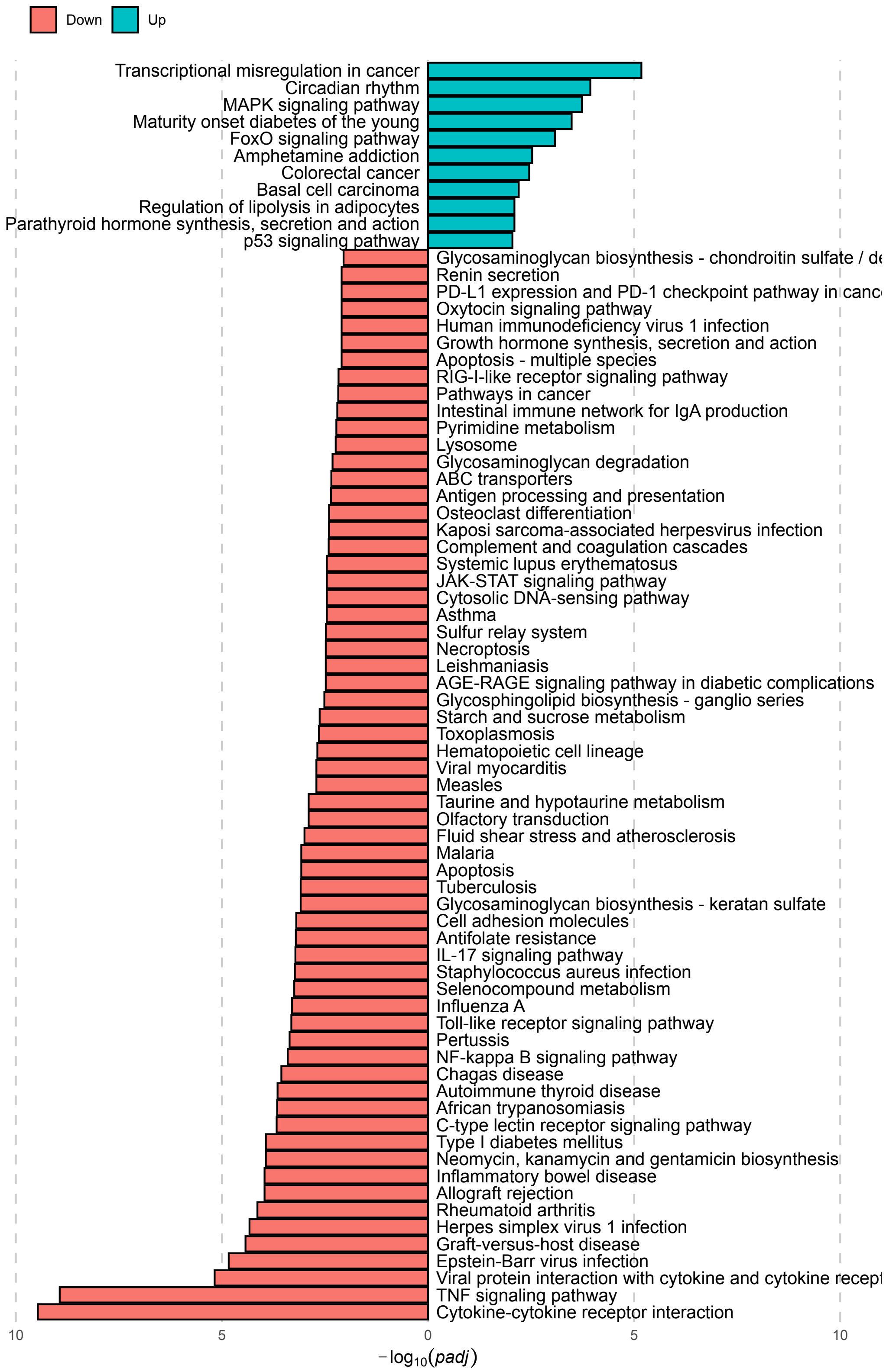

Supplementary Figure 2: Significant KEGG pathways involved in the inflammation process and differentially modulated by 3-oxo-C12:2-HSL were identified by EGSA method (p value<0.01). Red and green bars represent down-regulated and up-regulated pathways respectively.

Supplementary Figure 3:

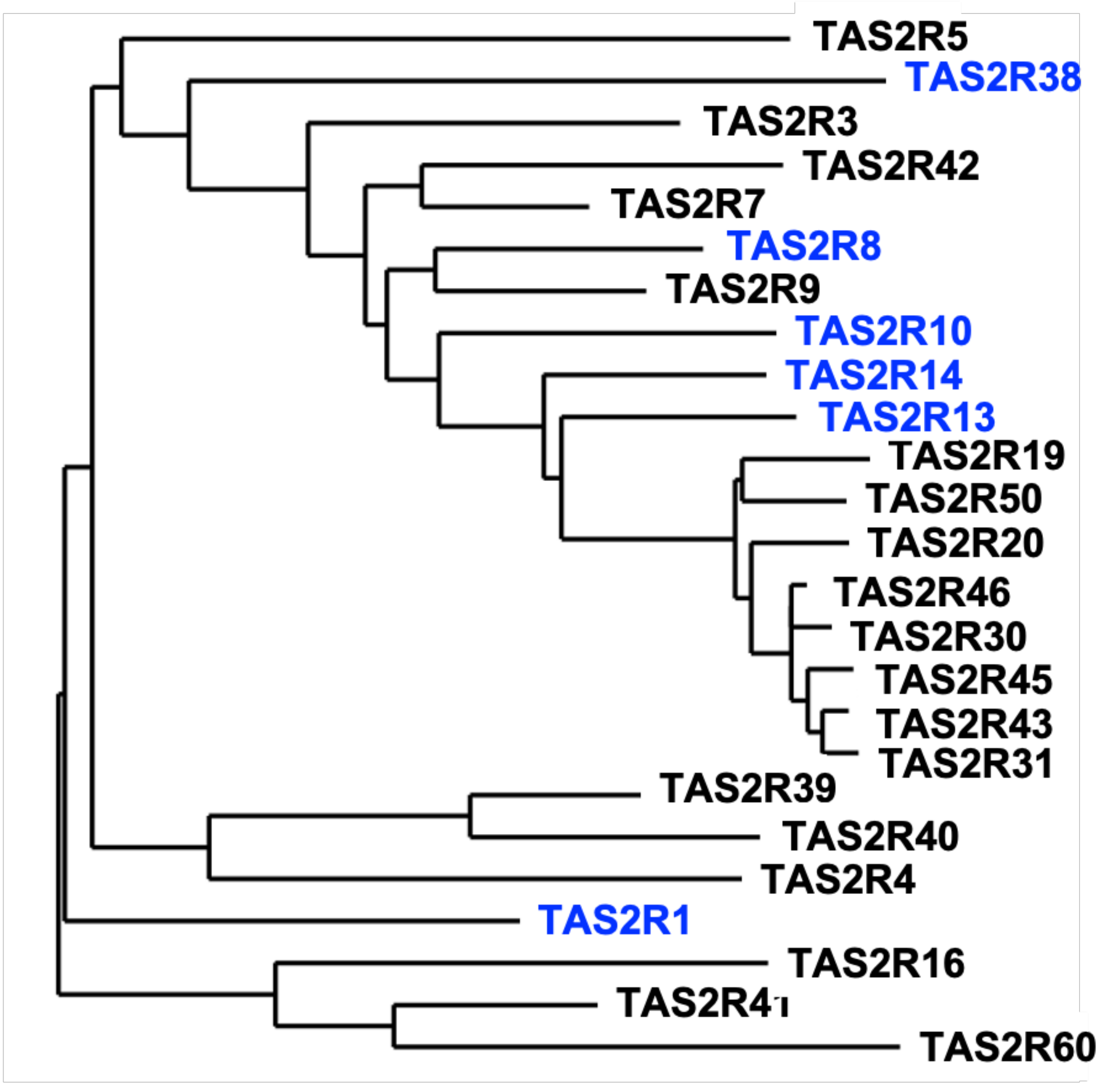

Supplementary Figure 3: Phylogenetic tree based on alignment of amino acid sequences of TAS2Rs. The neighbor-joining tree was constructed after a multiple sequence alignment of the TAS2Rs. The 6 TAS2R receptors responding to 3-oxo-C12:2-HSL are indicated in blue.
